# Expression Profile and Molecular Basis of Cyclin-Dependent Kinases Regulatory Subunit 2 in Endometrial Carcinoma Detected by Diversified Methods

**DOI:** 10.3389/pore.2022.1610307

**Published:** 2022-05-27

**Authors:** Li Gao, Gang Chen, Zi-Qian Liang, Jian-Di Li, Dong-Ming Li, Yu-Lu Tang, Deng Tang, Zhi-Guang Huang, Jun-Hong Chen, Jia-Yuan Luo, Jiang-Hui Zeng, Yi-Wu Dang, Zhen-Bo Feng

**Affiliations:** ^1^ Department of Pathology, The First Affiliated Hospital of Guangxi Medical University, Nanning, China; ^2^ Department of Pathology, Guangxi Maternal and Child Health Hospital, Nanning, China; ^3^ Department of Clinical Laboratory, The Third Affiliated Hospital of Guangxi Medical University/Nanning Second People’s Hospital, Nanning, China

**Keywords:** molecular mechanism, endometrial carcinoma, RNA-seq, CKS2, in-house tissue microarray

## Abstract

**Purpose:** Our purpose was to systematically appraise the clinicopathological significance and explore the molecular bases of CKS2 in endometrial carcinoma.

**Patients and Methods:** We measured the clinicopathological significance of CKS2 using diverse methods of public RNA-seq, microarrays, and in-house tissue microarrays to investigate the molecular basis of CKS2 in endometrial carcinoma through upstream transcriptional analysis, immune infiltration correlation analysis, and co-expression analysis.

**Results:** Both the analysis for public RNA-seq plus the microarray data and in-house tissue microarray confirmed the significant overexpression of CKS2 in a total of 1,021 endometrial carcinoma samples compared with 279 non-cancer endometrium samples (SMD = 2.10, 95% CI = 0.72–3.48). The upregulated CKS2 was significantly related to the lymph node metastasis and advanced clinical grade of endometrial carcinoma patients (*p* < 0.001). Mutation types such as amplification and mRNA occurred with high frequency in the CKS2 gene in endometrial carcinoma patients. A series of miRNAs and transcription factors, such as hsa-miR-26a, hsa-miR-130a, hsa-miR-30, E2F4, MAX, and GABPA, were predicted to regulate the transcription and expression of CKS2. Significant links were found between CKS2 expression and the infiltration level of B cells, CD4^+^ T cells, and neutrophils in endometrial carcinoma. CKS2-coexpressed genes were actively involved in pathways such as the mitotic cell cycle process, PID aurora B pathway, and prolactin signaling pathway.

**Conclusion:** The overexpressed CKS2 showed positive correlations with the clinical progression of endometrial carcinoma and was associated with various cancer-related biological processes and pathways, showing potential as a promising clinical biomarker for endometrial carcinoma.

## Introduction

As one of the most common malignancies of the female reproductive system, endometrial carcinoma caused over 417,000 newly diagnosed cases and over 97,000 deaths in 2020 [1]. In parallel with the increased rate of obesity and the growing aging female population, the incidence of endometrial carcinoma continues to rise [2, 3]. Based on clinical and histological characteristics, endometrial carcinoma was classified into type I and type II [4]. While obesity and high levels of estrogen were linked with type I endometrial carcinoma, which accounted for the majority of endometrial carcinoma and showed relatively good differentiation, type II endometrial carcinoma was found to be more aggressive [5]. In current times, surgical operation consolidated by adjuvant chemotherapy and radiotherapy serves as the main treatment modality for endometrial carcinoma patients [6–8]. Although great progress made in the therapy of endometrial carcinoma has remarkably curbed the progression of endometrial carcinoma, endometrial carcinoma patients in later stage are still tortured by worsening prognoses, and recurrences were reported in some patients in the early stage [9, 10]. Such a scenario highlights the imperative of seeking novel biomarkers and other treatment strategies.

Cyclin-dependent kinases regulatory subunit 2 (CKS2) is one of the components of the cycle-dependent protein kinase subunits family that shows high conservatism in eukaryotes and plays essential roles in the regulation of the cell cycle of somatic cells and early embryogenesis [11, 12]. A growing body of evidence has indicated that CKS2 exhibited upregulation in a variety of human cancers and contributed to tumor progression [12–14]. CKS2 was reported to be overexpressed in cholangiocarcinoma and stimulate the aggressiveness of cholangiocarcinoma by modulating cell cycle progression and mitochondrial caspase-dependent apoptosis [15]. In esophageal squamous cell carcinoma, CKS2 expression was significantly higher in cancer tissues, and suppression of CKS2 attenuated the growth of esophageal squamous cell carcinoma [16]. So far, there has been no research on the overall assessment of the clinicopathological significance of CKS2 in endometrial carcinoma and the molecular basis of CKS2 in the oncogenesis of endometrial carcinoma.

Herein, we planned to systematically appraise the clinicopathological significance and explore the molecular bases of CKS2 in endometrial carcinoma using multiple detection technologies, including microarrays, RNA-seq, and in-house tissue microarrays.

## Materials and Methods

### Evidence From Public Microarrays and RNA-Seq Datasets

Clinical data of endometrial carcinoma patients and gene expression values (in the data format of fragments per kilobase per million or transcripts per kilobase million) in endometrial carcinoma and non-cancer endometrium tissues were imported from the Genotype-Tissue Expression (GTEx) project and The Cancer Genome Atlas (TCGA) database. Detailed clinicopathological information on endometrial carcinoma patients from the TCGA database is listed in Supplementary Table S1. The incorporated dataset of the TCGA-GTEx expression matrix (containing 552 endometrial carcinoma and 177 non-cancer endometrium samples) was normalized by the formula of log2 (transcripts per kilobase million value +0.001). Other sources for our study’s expression analysis (Table 1) were microarray datasets in the Gene Expression Omnibus (GEO) or ArrayExpress databases, which deposit the gene expression matrix of at least three endometrial carcinoma and three non-cancer endometrium samples that belong to the human species (before 7 June 2021).

**TABLE 1:** Basic information from all included RNA-seq and microarray datasets of endometrial carcinoma.

Accession ID	Platform	Country	First author	Sample type	Number of tumor samples	Number of non-cancer samples	Experiment type	Histological type
GSE17025	GPL570	United States	Uma Chandran	Tissue	91	12	Expression profiling by array	Endometrioid endometrial carcinoma and serous endometrial carcinoma
GSE56087	GPL11154	China	Yifeng Zhou	Tissue	9	9	Expression profiling by high throughput sequencing	Not specified
GSE63678	GPL571	United States	Prokopios Alexandros Polyzos	Tissue	7	5	Expression profiling by array	Not specified
GSE115810	GPL96	Poland	Urszula Mazurek	Tissue	24	3	Expression profiling by array	Not specified
GSE146889	GPL16791	United States	Asha Nair	Tissue	37	35	Expression profiling by array	Not specified
TCGA-GTEx	—	—	—	Tissue	552	177	—	Endometrioid endometrial adenocarcinoma, Serous endometrial adenocarcinoma and mixed serous and endometrioid

### Expression Analysis for CKS2 Utilizing External RNA-Seq and Microarray Datasets

We conducted the extraction and processing of CKS2 expression data according to the methods in prior works [17]. Microarrays were aggregated by the GPL platform, and the batch effect was removed for microarrays from the same platform through the limma package loaded by R software v.3.6.1. The standard mean difference (SMD) plot was calculated for comparison of the different expressions of CKS2 in endometrial carcinoma versus non-cancer tissues, and the corresponding summarized receiver operating characteristics (SROC) curves were plotted. The steps of drawing SMD forest plots and SROC curves and be found in previous studies [18]. The expression pattern of CKS2 in endometrial carcinoma and the distinguishing ability of CKS2 expression for endometrial carcinoma were exhibited as violin plots and ROC curves for all included RNA-seq datasets and public microarrays [19, 20].

### In-House Tissue Microarray

Surgical resected samples of 301 endometrial carcinoma tissues (two undifferentiated carcinoma cases, 295 endometrial endometrioid adenocarcinoma tissues, and 4 endometrioid adenocarcinoma with squamous differentiation tissues) and 38 normal endometrium tissues (date: January 2018 to June 2019) provided by Guilin Fanpu Biotech of Guangxi, China, were enrolled for the immunohistochemistry (IHC) experiment. The basic information for all included tissues is included in Supplementary Table S2. Informed consent was provided by all involved endometrial carcinoma patients, and the ethics committee of Guilin Fanpu Biotech and that of the First Affiliated Hospital of Guangxi Medical University [Approval ID: 2020 (KY-E-095)] authorized the study.

Firstly, the paraffin section was put into a 60°C oven for 120 min followed by routine dewaxing of paraffin sections. After being soaked in different concentrations of ethanol, the slides were rinsed with tap water for 3 min. Antigen repair was conducted on the slides with EDTA antigen repair solution (distilled water: EDTA = 50:1). Endogenous peroxidase blocker (hydrogen peroxide 30%, CAS 7722-84-1, Chengdu Kelong Chemical Co., Ltd.) (100 μl) was applied to each slide at room temperature and washed by PBS following 10 min’ incubation. The antibody for CKS2 (https://www.abcam.cn/cks2-antibody-epr79462-ab155078.html) and an enzyme labeled Sheep anti-rabbit IgG polymer (secondary antibody) were used for two periods of incubation. Subsequently, incubation with hematoxylin staining solution and gradient alcohol dehydration was performed. Finally, the slides were made transparent by xylene and sealed with neutral gum and coverslip. Two pathologists reviewed and assessed the immunostaining of all images under a microscope (Motic China Group Co., Ltd.) (magnification, ×200 and ×400). The scores for percentage of positively stained cells (0, <5%; 1, 5%–25%; 2, 26%–50%; 3, 51%–75%; 4, >75%) multiplied by scores for staining intensity (0, negative; 1, weak; 2, medium; 3, strong) were the final IHC scores of each slide. The range of possible scores was 0–12. An IHC score of <4 indicated low CKS2 protein expression and an IHC score of ≥4 indicated high CKS2 protein expression.

### Survival Analysis for CKS2 in Endometrial Carcinoma

To investigate whether CKS2 expression has an impact on the overall survival of endometrial carcinoma patients or not, we plotted Kaplan-Meier survival curves using GraphPadPrism v.8.0.1 based on information from 535 endometrial carcinoma patients without adjuvant treatment from RNA-seq datasets in TCGA database. The accompanied log-rank test was performed and *p* < 0.05 means statistically significant.

### Landscape of Genetic Mutation of CKS2 in Endometrial Carcinoma

We made use of the cBioPortal database to query mutation types and z-scores of mRNA expression (log RNA Seq V2 RSEM) of CKS2 in 548 endometrial carcinoma samples using genomic data from the GDAC Firehose project.

### Potential Up-Stream Regulatory Mechanism of CKS2

By considering the critical roles of miRNA and transcription factor (TF)s in regulating gene expression, we explored the regulatory mechanism of aberrant CKS2 expression from the aspects of miRNA and TFs. Upstream miRNAs and TFs that might control the transcription and expression of CKS2 were estimated by the miRWalk 3 and NetworkAnalyst databases.

### Correlation Between CKS2 Expression and Immune Infiltration in Endometrial Carcinoma

The online tool Tumor Immune Estimation Resource (TIMER) was employed for evaluating the correlation between CKS2 expression and the infiltration level of diversified immune cells including B cells, T cells, Neutrophils, Macrophages, and Dendritic cells. Significant relationships were filtered by a *p* value of 0.05.

### Characterization of Molecular Function for CKS2-Co-Expressed Genes in Endometrial Carcinoma

We carried out differential expression analysis to compare gene expression profiles of endometrial carcinoma and non-cancer endometrium tissues from all included microarrays via limma package loaded by R software v.3.6.1. Differentially expressed genes (DEGs) in the RNA-seq dataset were calculated with a count matrix using the voom algorithm in R software v.3.6.1. DEGs of endometrial carcinoma met the conditions of showing significant upregulation (log2FC > 1, adj. *p* < 0.05) or significant downregulation (log2FC < −1, adj. *p* < 0.05) in at least three datasets. The associations between expression values of genes were appraised via a Pearson correlation test embedded in the psych package loaded by R software v.3.6.1. Upregulated DEGs that were positively correlated with the expression of CKS2 (r > 0, adjusted *p* < 0.05) in four or more datasets of endometrial carcinoma were regarded as genes positively related to CKS2 in endometrial carcinoma; genes negatively correlated with CKS2 in endometrial carcinoma were downregulated DEGs that showed negative relationship with CKS2 (adjusted *p* < 0.05, r < 0) in three or more datasets. Functional enrichment of the above genes with significant relations to CKS2 was annotated through pathway analysis and gene ontology (GO). Functional annotation was conducted with MetaScape and adjusted *p* < 0.05 was the cutoff value for significant pathway and GO terms.

### Statistical Analysis

SPSS 22.0 was applied for analyzing the clinicopathological significance of CKS2 in endometrial carcinoma samples from the RNA-seq dataset and in-house tissue microarrays. At last, statistical significance was indicated by *p* < 0.05. The Independent sample *t*-test was used for the comparison of CKS2 expression in two groups of clinicopathological variables. Comparison of CKS2 expression in three or more groups of clinicopathological variables was conducted through analysis of variance or Kruskal-Wallis test when normal distribution was not met.

## Results

### Distinct CKS2 Expression Between Endometrial Carcinoma and Non-Cancer Endometrium Tissues

**FIGURE 1:**
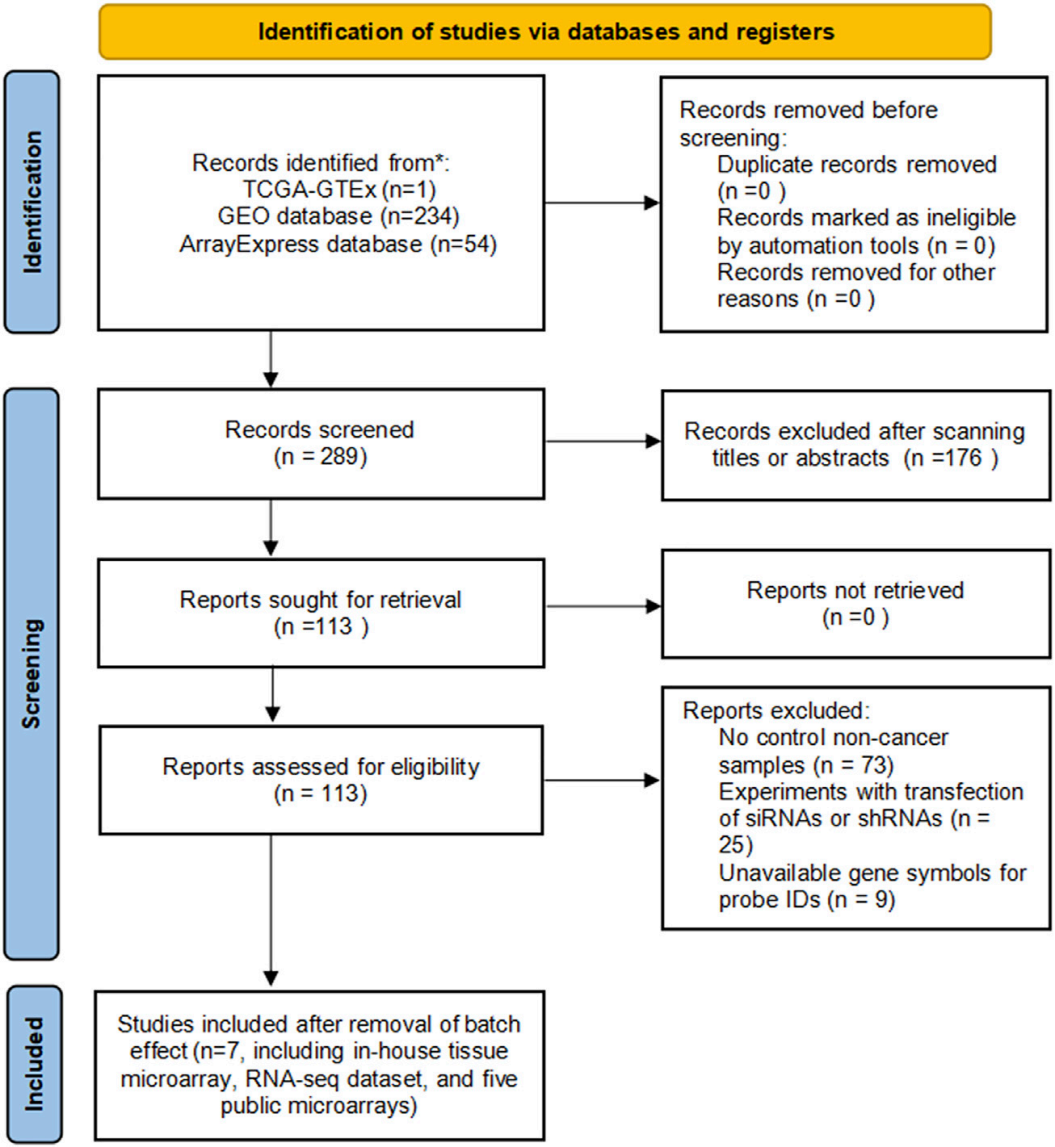
Flowchart of inclusion of eligible microarrays and RNA-seq datasets.

**FIGURE 2:**
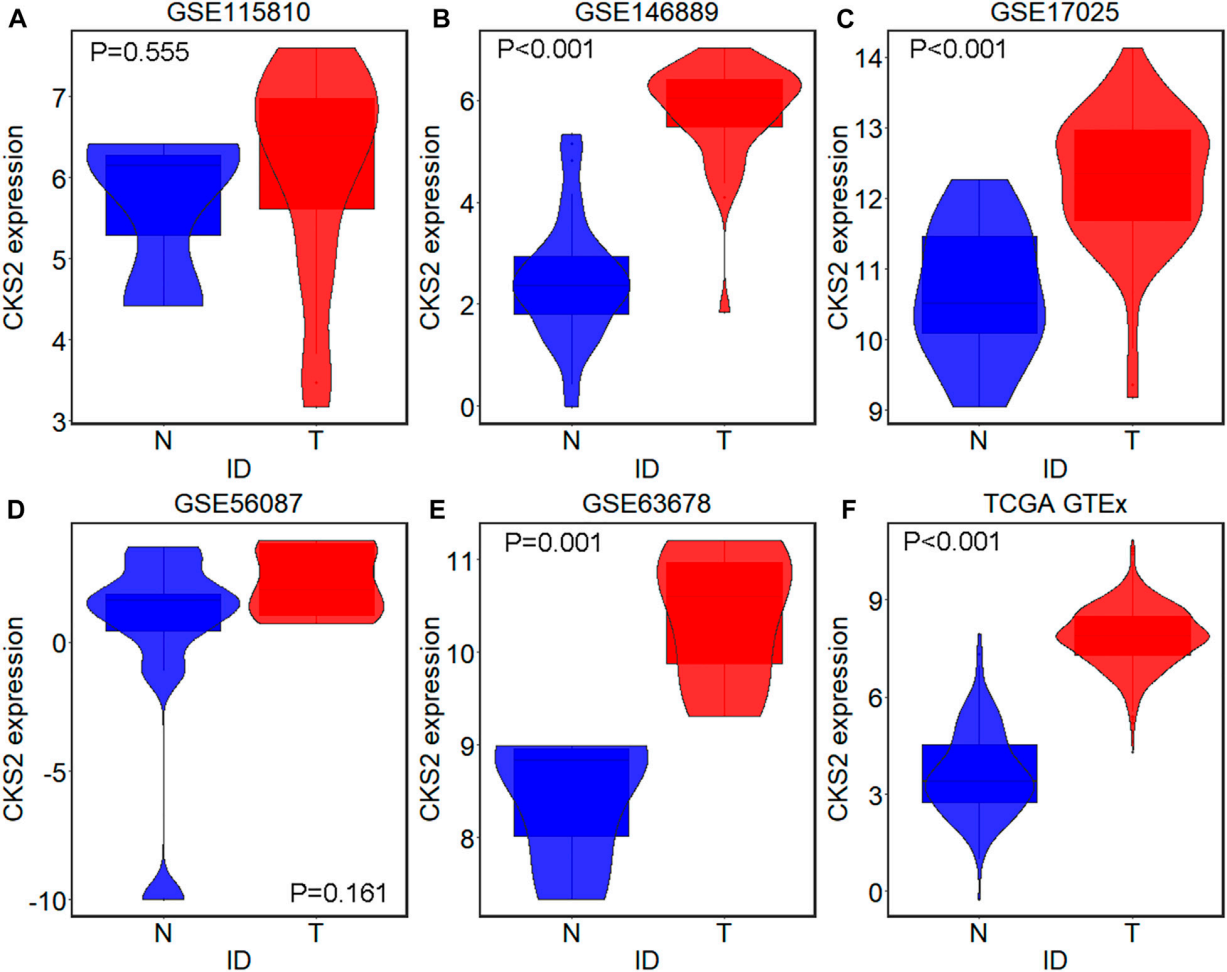
CKS2 expression in endometrial carcinoma and non-cancer samples from external microarrays and RNA-seq dataset. Violin plots for GSE115810 **(A)**, GSE146889 **(B)**, GSE17025 **(C)**, GSE56087 **(D)**, GSE63678 **(E)** and TCGA-GTEx dataset **(F)**. N, non-cancer endometrium samples; T, endometrial carcinoma samples.

**FIGURE 3:**
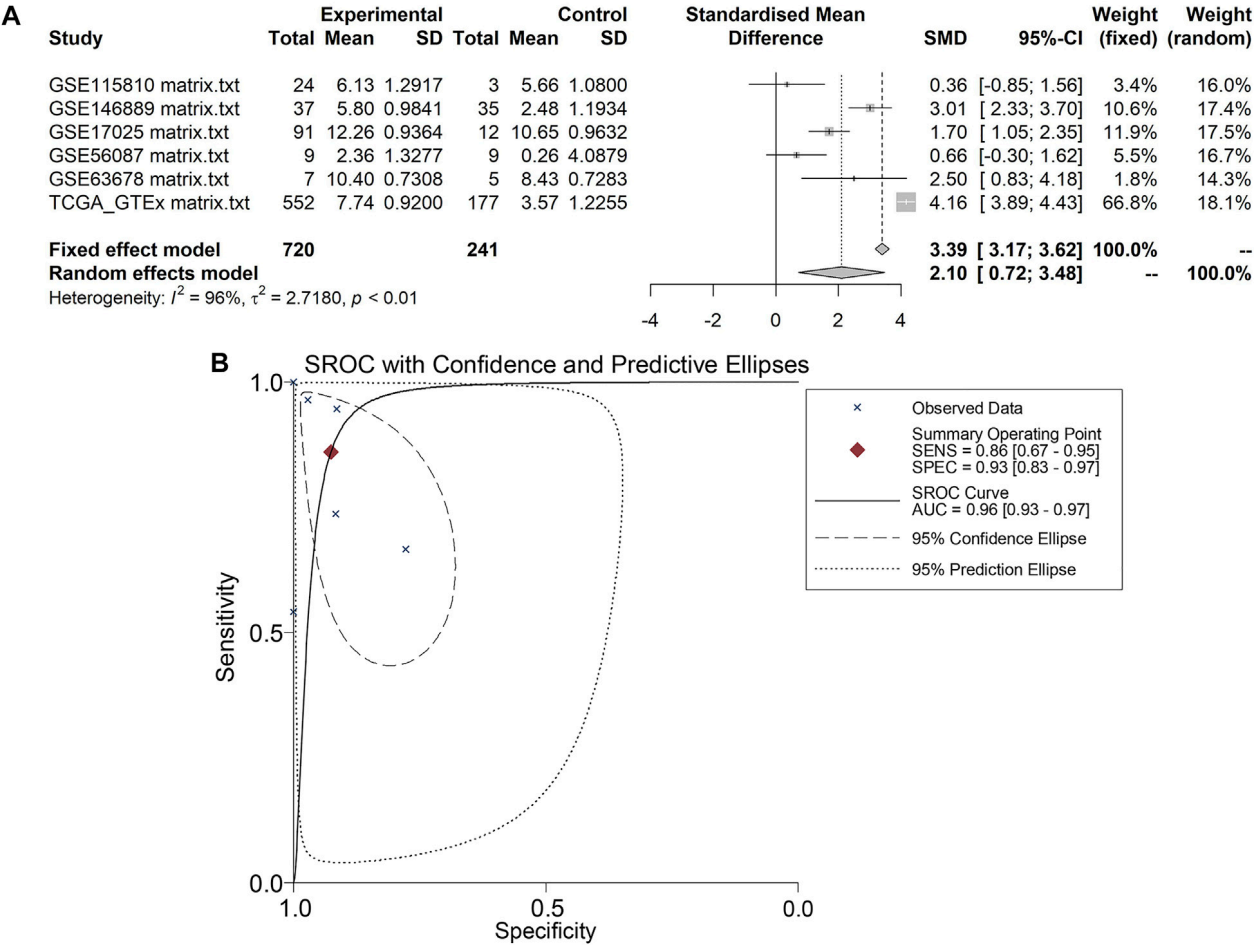
Pooling results of CKS2 expression in endometrial carcinoma and non-cancer tissues for in-house tissue microarray, public microarrays, and RNA-seq datasets. **(A)** SMD forest. **(B)** SROC curves.

**FIGURE 4:**
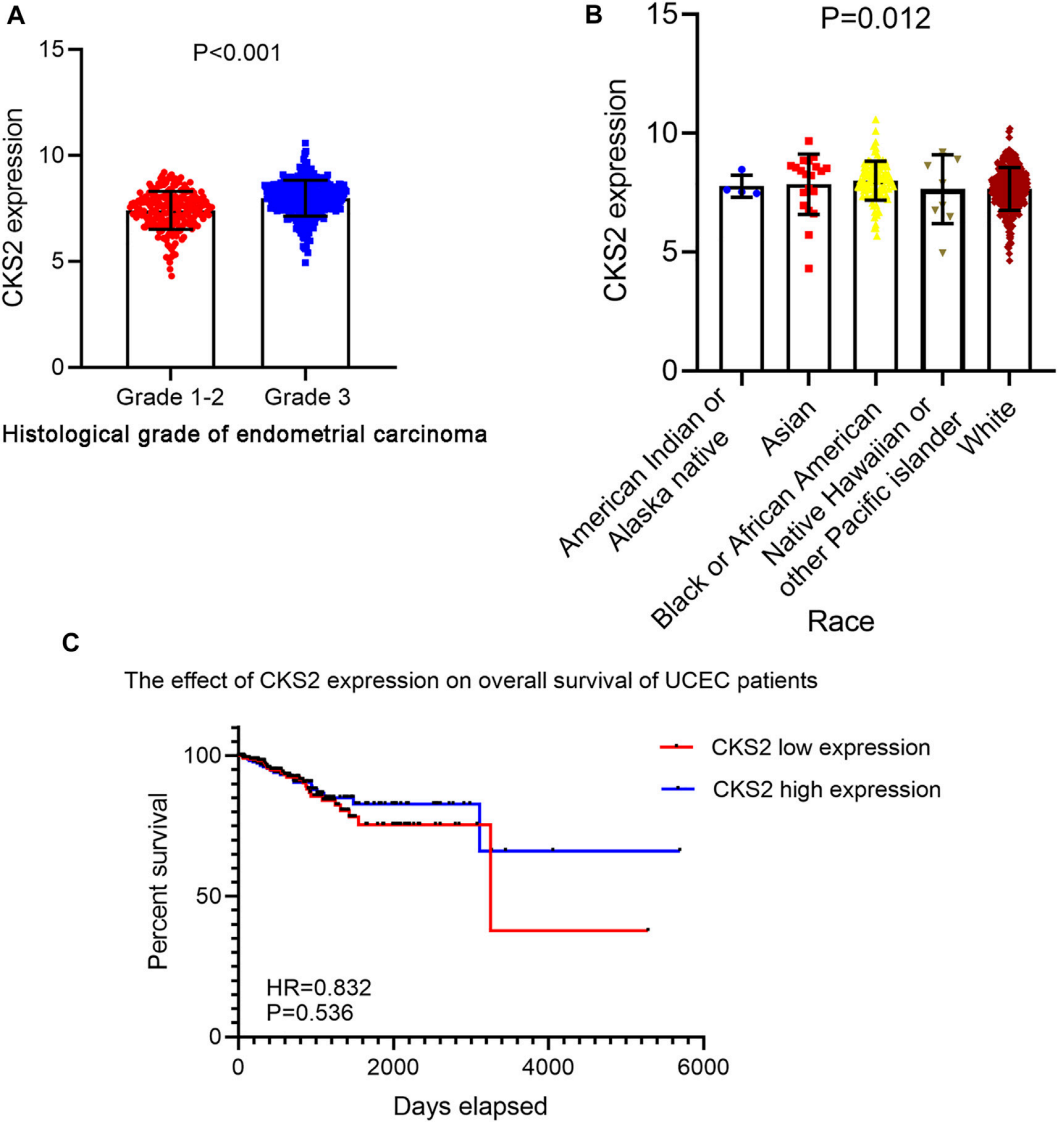
The clinicopathological significance of CKS2 expression in endometrial carcinoma from the RNA-seq dataset. **(A)** The remarkable differential expression of CKS2 between endometrial carcinoma patients of lower grade and higher grade. **(B)** Expression pattern of CKS2 in different race groups of endometrial carcinoma patients. **(C)** Kaplan-Meier survival curves of the overall survival probability of endometrial carcinoma patients.

A total of five microarrays after the removal of batch effect and TCGA-GTEx RNA-seq datasets were included for expression analysis of CKS2 in endometrial carcinoma, the selection process of which was illustrated in Figure 1. Clinical information of all endometrial carcinoma patients from the RNA-seq dataset in the TCGA database and details of all included datasets are described in Supplementary Table S1 and Table 1. All endometrial carcinoma and non-cancer samples from public microarrays and RNA-seq datasets amounted to 720 and 241, respectively. CKS2 displayed obvious higher expression in endometrial carcinoma and a favorable distinguishing ability for endometrial carcinoma in the majority of datasets (Figure 2) (Supplementary Figure S1). SMD forest plot and SROC curves generated from all included datasets confirmed the overexpression of CKS2 in endometrial carcinoma and the capacity of upregulated CKS2 in discerning endometrial carcinoma from non-cancer endometrium tissues (SMD = 2.10, 95% CI = 0.72–3.48; AUC = 0.96) (Figure 3). Analysis of the relationships between CKS2 expression and the clinicopathological variables of endometrial carcinoma patients from the RNA-seq dataset in the TCGA database reported higher CKS2 expression in endometrial carcinoma patients with a higher grade (G3) and Black or African American patients (*p* < 0.001) (*p* = 0.012) (Figures 4A,B) (Table 2).

**TABLE 2:** The relationship between CKS2 expression and the clinicopathological features of endometrial carcinoma patients from the RNA-seq dataset in the TCGA database.

Clinical features	CKS2 expression value			*t*	*p*-value
	Number	Mean	Standard deviation		
Menopause status					
Inter-menopause	17	7.545	0.956	1.317^a^	0.268
Peri-menopause	17	7.360	0.829		
Post-menopause	445	7.763	0.904		
Pre-menopause	35	7.723	1.146		
BMI					
<30	209	7.795	0.896	1.312	0.190
≥30	303	7.688	0.918		
Race					
American Indian or Alaska native	4	7.769	0.467	12.953^b^	0.012
Asian	20	7.852	1.268		
Black or African American	106	8.002	0.814		
Native Hawaiian or other Pacific islander	9	7.644	1.449		
White	372	7.659	0.899		
Ethnicity					
Hispanic or Latino	15	7.751	1.130	0.046	0.963
Not Hispanic or Latino	373	7.740	0.926		
Neoplasm cancer status					
Tumor free	427	7.706	0.904	-1.484	0.138
With tumor	78	7.876	1.055		
Histological type					
Endometrioid endometrial adenocarcinoma	407	7.695	0.948	4.539^b^	0.103
Mixed serous and endometrioid	22	7.742	0.815		
Serous endometrial adenocarcinoma	114	7.946	0.793		
Age					
<60	178	7.771	0.992	0.372	0.710
≥60	365	7.739	0.879		
Peritoneal wash					
Negative	350	7.694	0.922	−0.696	0.487
Positive	57	7.786	0.919		
Histologic grade					
G1-2	218	7.406	0.905	−7.514	<0.001
G3	325	7.980	0.852		
Residual tumor					
R0	372	7.735	0.956	0.042^a^	0.959
R1	22	7.741	1.031		
R2	16	7.665	0.733		
Venous invasion					
No	40	4.477	1.662	−0.945	0.486
Yes	63	4.829	1.948		
History of birth control pill usage					
No	48	4.545	1.654	−1.084	0.280
Yes	100	4.883	1.835		
Clinical stage					
I-II	390	7.703	0.925	−1.913	0.056
III-IV	153	7.870	0.887		

a Analysis of variance was performed.

b Kruskal-Wallis test was performed.

**FIGURE 5:**
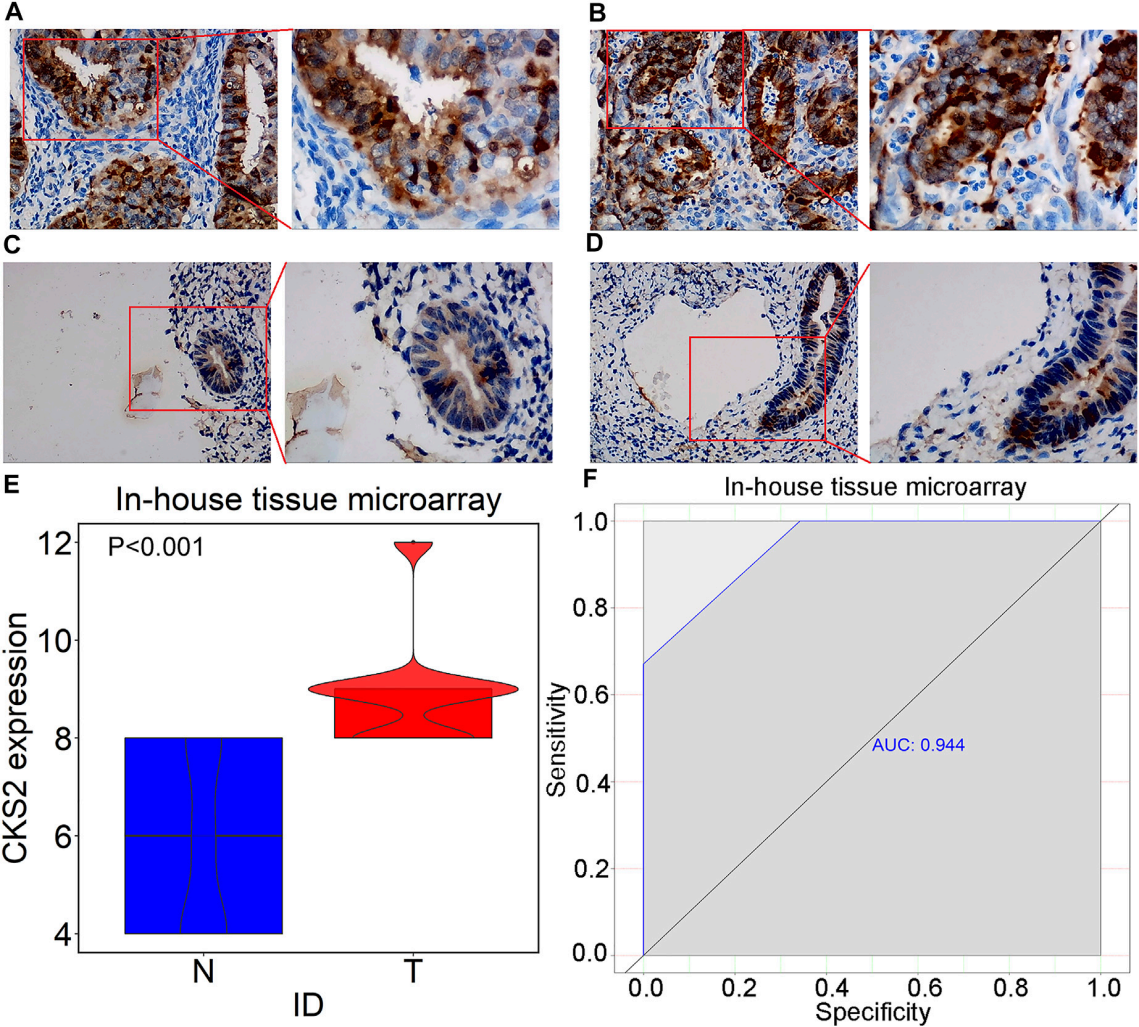
IHC staining of CKS2 in endometrial carcinoma and non-cancer tissues from in-house tissue microarrays. **(A)** Strong staining of CKS2 in endometrial carcinoma tissues (Left: ×200; Right: ×400); **(B)** Strong staining of CKS2 in endometrial carcinoma tissues (Left: ×200; Right: ×400); **(C)** Weak staining of CKS2 in non-cancer endometrium tissues (Left: ×200; Right: ×400); **(D)** Weak staining of CKS2 in non-cancer endometrium tissues (Left: ×200; Right: ×400); **(E)** Violin plot of CKS2 expression in endometrial carcinoma and non-cancer endometrium tissues. **(F)** ROC curves of the discriminating ability of CKS2 expression for endometrial carcinoma. N, non-cancer endometrium samples. T, endometrial carcinoma samples.

Raw data related to the endometrial carcinoma patients included in tissue microarray was listed in Supplementary Table S2. As shown by the IHC staining results for 301 endometrial carcinoma and 38 non-cancer endometrium tissues, CKS2 expression was remarkably higher in the glandular epithelial cells of endometrial carcinoma tissues than in non-caner endometrium tissue (9.071 ± 1.122; 5.789 ± 1.788, *p* < 0.001) (Figure 5). The differentially expressed CKS2 also showed good performance in discriminating endometrial carcinoma from non-cancer endometrium tissues (AUC = 0.944) (Figure 5H). Moreover, endometrial carcinoma patients with lymph node metastasis presented notably higher CKS2 expression than endometrial carcinoma patients without lymph node metastasis (*p* < 0.001) (Supplementary Figure S2) (Table 3).

**TABLE 3:** The relationship between CKS2 expression and the clinicopathological features of endometrial carcinoma patients from in-house tissue microarray.

Clinical features	CKS2 expression value			*t*	*p*-value
	Number	Mean	Standard deviation		
Pathological classification					
Endometrioid adenocarcinoma	295	8.99	1.131	0.369^a^	0.691
Endometrioid adenocarcinoma with squamous differentiation	4	8.50	0.577		
Undifferentiated carcinoma	2	9.00	0.000		
Age					
<60	238	8.92	1.088	−1.326	0.186
≥60	58	9.14	1.146		
Histologic grade					
G1-2	207	8.96	1.072	−0.385	0.701
G3	83	9.01	1.205		
T stage					
1–2	285	8.94	1.094	−2.098	0.052
3–4	16	9.69	1.401		
Lymph node metastasis					
No	260	8.73	0.724	−6.779	<0.001
Yes	41	10.59	1.732		

a Analysis of variance was performed.

### Influence of CKS2 Expression on the Prognosis of Endometrial Carcinoma Patients

Kaplan-Meier survival analysis for 535 endometrial carcinoma patients without adjuvant treatment in RNA-seq dataset of TCGA database indicated no significant impact of CKS2 expression on the survival time of endometrial carcinoma patients (hazard ratio (HR) = 0.832, *p* = 0.536) (Figure 4C).

### Genetic Mutation Types of CKS2 in Endometrial Carcinoma

Four cases of amplification, four cases of deep depletion, three cases of mRNA high, and seven cases of mRNA low were displayed in the genetic alteration profiles of 548 endometrial carcinoma cases from the GDAC Firehose project of cBioPortal (Supplementary Figure S3).

### Prediction of Upstream miRNAs and TFs for CKS2

A series of miRNAs such as has-miR-26hashsa-miR-130a, and hsa-miR-30c as well as TFs, including E2F4, MAX, and GABPA, emerged in the networks with the potential to regulate the transcription of CKS2 (Supplementary Figure S4).

### Correlation Between CKS2 Expression and Immune Infiltration in Endometrial Carcinoma

**FIGURE 6:**
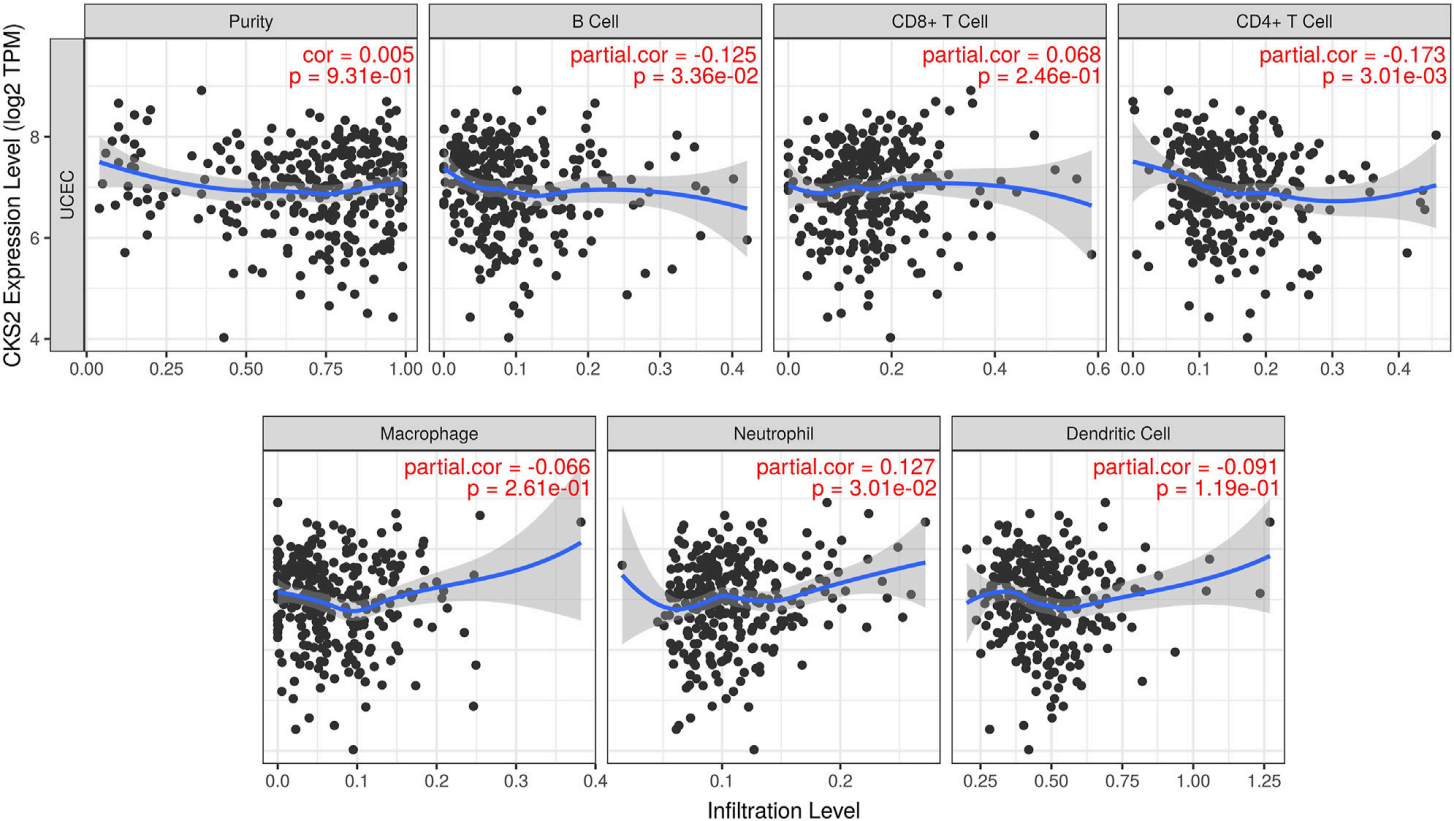
The relationship between CKS2 expression and the tumor purity or infiltration level of diverse immune cells in endometrial carcinoma. TPM: transcripts per million.

Pearson correlation analysis results revealed that CKS2 expression was negatively linked with the infiltration level of B cell and CD4^+^ T cell in endometrial carcinoma (*r* = −0.125, *p* = 0.034; *r* = −0.173, *p* = 0.003). A positive relationship was found between CKS2 expression and the infiltration level of neutrophils (*r* = 0.127, *p* = 0.030) (Figure 6).

### Molecular Functions of CKS2-Related Genes in Endometrial Carcinoma

**FIGURE 7:**
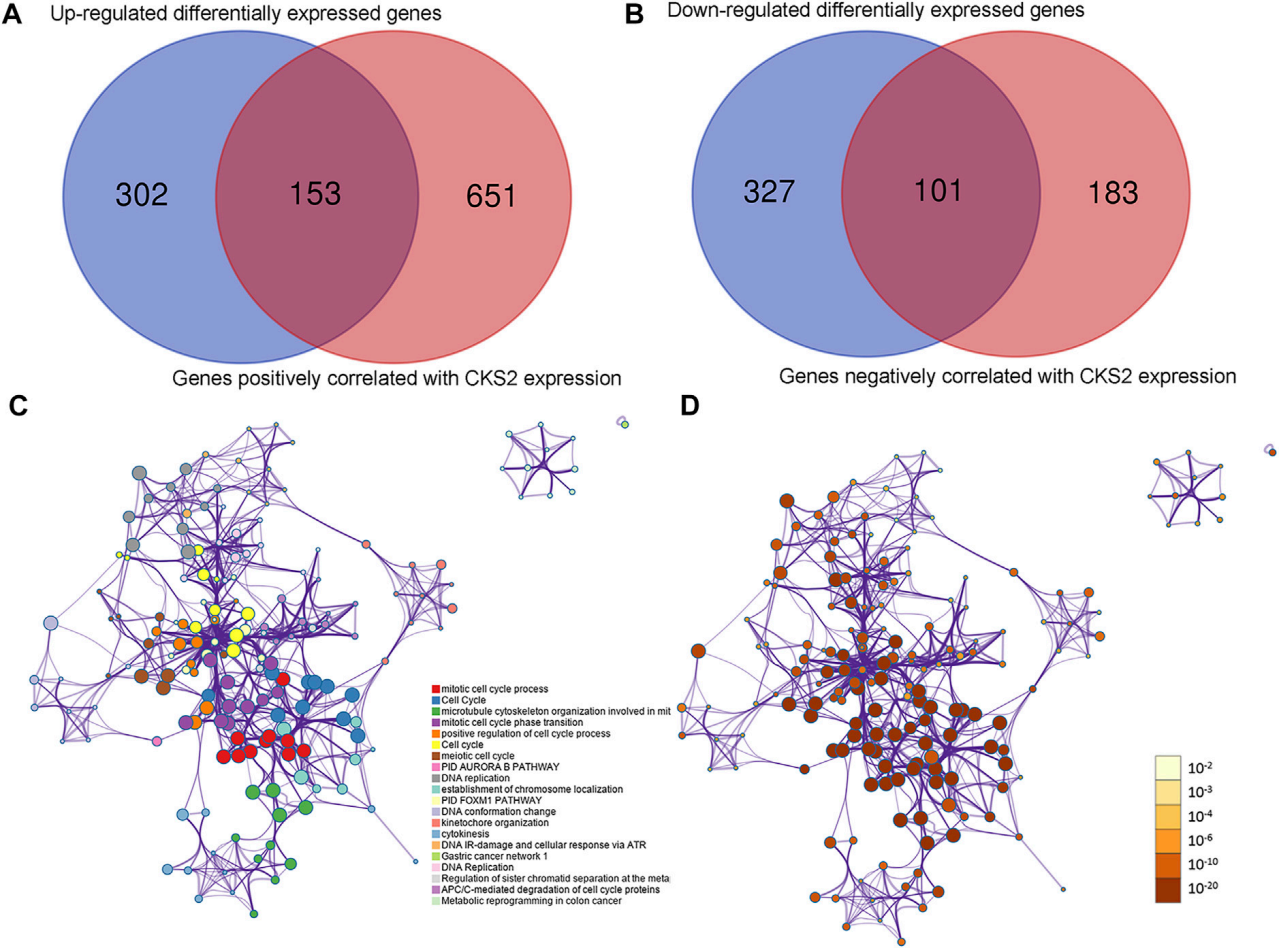
Co-expressed genes of CKS2 and functional annotations for genes positively correlated with CKS2 in endometrial carcinoma. **(A)** Venn plot for genes positively co-expressed with CKS2 in endometrial carcinoma. **(B)**. Venn plot for genes negatively co-expressed with CKS2 in endometrial carcinoma. **(C)**. Network of enriched terms for positively co-expressed genes colored by cluster ID. **(D)**. Network of enriched terms for positively co-expressed genes colored by *p*-value.

Of 455 identified upregulated DEGs and 428 identified downregulated DEGs in endometrial carcinoma, 153 genes positively co-expressed with CKS2 and 101 genes negatively co-expressed with CKS2 were screened out (Figure 7A). Genes positively co-expressed with CKS2 were annotated to cluster in biological processes and pathways including mitotic cell cycle process, microtubule cytoskeleton organization involved in mitosis, and PID aurora B pathway (Figure 7B). Biological process and pathway terms such as the circulatory system process, calcium ion transport, and the prolactin signaling pathway were prominently assembled by genes negatively co-expressed with CKS2 (Supplementary Figure S5).

## Discussion

The flourishing of genome sequencing and high-throughput microarrays generated huge amounts of data useful for oncological research. Making full use of big data and in-house molecular pathology technology, we were the first group to measure the clinicopathological significance of CKS2 in pooled multi-center endometrial carcinoma samples and to investigate the molecular basis of CKS2 in endometrial carcinoma through upstream transcriptional analysis, immune correlation analysis, and co-expression analysis.

First of all, an incorporated expression matrix from public RNA-seq and microarrays as well as data from in-house tissue microarray uniformly supported the overexpression of CKS2 in endometrial carcinoma. The reliability of the study is embodied in the large sample size of 1,021 endometrial carcinoma cases and 279 non-cancer endometrium cases. The significant associations between CKS2 expression and lymph node metastasis or grade of endometrial carcinoma revealed by independent sample *t* test with the clinical-pathological parameters implied the positive links between CKS2 overexpression and the clinical progression of endometrial carcinoma. The prognostic value of CKS2 has been discovered in multiple human cancers including adrenocortical carcinoma, lung adenocarcinoma, and colorectal cancer [21–23]. However, the survival analysis in this study suggested no significant influence of CKS2 expression on prognosis of endometrial carcinoma patients; thus, whether CKS2 expression carries a prognostic value for predicting the survival time of endometrial carcinoma patients needed to be explored with larger cohorts in future studies.

Despite the fact that the carcinogenesis of endometrial carcinoma complicated genetic interactions and activities of pathways, finding an appropriate entry point would be conducive to the exploration of the mechanisms. In the present work, we investigated the molecular mechanisms of CKS2 in endometrial carcinoma via diverse methods of genetic mutation profile analysis, upstream transcriptional analysis, immune correlation analysis, and co-expression analysis. It could be noted from the overview diagram of the genetic alteration types of CKS2 in endometrial carcinoma that amplification and mRNA high constituted the half of cases with mutation of the CKS2 gene, which might interpret the upregulation of CKS2 in endometrial carcinoma and other human cancers. In view of the critical roles of upstream regulators such as miRNAs and TFs in modulating the transcription and expression of genes, we also built the miRNAs/TFs-CKS2 networks to probe into the CKS2-centered transcriptional regulatory mechanisms. The novelty of findings in this part lies in that the interactions between CKS2 and most of the miRNAs or TFs predicted above have not been reported by research. It could be speculated that these proposed miRNAs and TFs may serve as the initiators of the oncogenic effect of CKS2 in endometrial carcinoma. With regard to the correlation between CKS2 expression and the infiltration level of immune cells in endometrial carcinoma, significant bonds were observed between CKS2 expression and the immune infiltration of B cells, CD4^+^ T cells, or neutrophils. It has been reported that patients with polymerase ε ultra-mutated endometrial carcinomas had better prognosis than patients with polymerase ε-wild type endometrial carcinoma and this phenomenon was linked with the activated CD4^+^ T cell responses in polymerase ε ultra-mutated endometrial carcinoma patients [23], from which it could be inferred that the enhanced responses of CD4^+^ T cells might mitigate the malignant development of endometrial carcinoma. We assumed that upregulated CKS2 might reshape the tumor microenvironment of endometrial carcinoma by means of inhibiting the population of B cells and CD4^+^ T cells or increasing the population of neutrophils to contribute to the initiation and progression of endometrial carcinoma.

Lastly, we identified the CKS2-correlated genes in endometrial carcinoma via rigorous calculation of DEGs and correlation expression analysis. Functional annotation for the CKS2-correlated genes revealed a range of interesting biological processes and pathway terms. There were obvious variations between biological processes enriched by genes positively correlated with CKS2 and biological process genes negatively correlated with CKS2. While terms associated with cell cycle and DNA replication appeared with high frequency in the enrichment results for genes positively co-expressed with CKS2, the biological process or pathway terms enriched by genes negatively co-expressed with CKS2 covered various aspects of cellular functions, including migration, apoptosis, and integrin activation, from which it could be conjectured that the genes positively or negatively correlated with CKS2 may affect the CKS2-centered occurrence and progression of endometrial carcinoma through different biological processes and pathways. Particularly, the impact of CKS2 expression on biological processes such as cell cycle regulation and migration of tumor cells has been verified in other human cancers. The study of Gao et al. demonstrated the G2/M arrest of tongue squamous cell carcinoma cells induced by inhibition of CKS2 expression, and the regulatory effect of CKS2 on the cell cycle progression of tongue squamous cell carcinoma cells was linked with the interaction between CKS2 and DUTPase [24]. Another research on hepatocellular carcinoma unveiled the suppression of knocking down CKS2 expression on the migratory ability of hepatocellular carcinoma cells [25]. The mechanistic bridge between upregulated CKS2 and carcinogenesis of human cancers also involved DNA damage responses and DNA replication. The work of Liberal et al. showed that overexpression of CKS1 or CKS2 in human cancer cells triggered override of the DNA replication blockage under the circumstances of replication stress, enabling DNA replication of cancer cells and thus conferring the advantages in proliferation to boost the development of tumors [26, 27]. The recorded enrichment of CKS2-correlated genes in the above pathways might serve as an explanation for the oncological mechanisms of CKS2 overexpression in endometrial carcinoma.

Aside from the encouraging findings of these works, we were also aware of the limitations of the present study. *In vitro* and *in vivo* experiments should be carried out in future work to validate the functional roles of CKS2 in the development of endometrial carcinoma and the genetic interaction of CKS2 with co-expressed genes.

## Conclusion

In conclusion, we identified the upregulation of CKS2 in endometrial carcinoma with tissue microarrays, RNA-seq, and other public microarrays. The upregulated CKS2 was significantly related to the clinical progression of endometrial carcinoma patients. The oncogenic effect of CKS2 in endometrial carcinoma might be driven by the abnormal miRNA/TF mediated upstream transcriptional regulation and activities of co-expressed genes in certain molecular functions or pathways.

## Data Availability

The datasets presented in this study can be found in online repositories. The names of the repository/repositories and accession number(s) can be found in the article/Supplementary Material.

## Supplementary Material

Supplementary Figure S1 The discriminatory ability of CKS2 expression in discerning endometrial carcinoma from non-cancer endometrium tissues. ROC curves for GSE115810 **(A)**, GSE146889 **(B)**, GSE17025 **(C)**, GSE56087 **(D)**, GSE63678 **(E)** and TCGA-GTEx dataset **(F)**. AUC, area under the curve.

Supplementary Figure S2 The notable relationships between CKS2 expression and clinicopathological features of endometrial carcinoma patients from an in-house tissue microarray cohort. **(A)** Violin plot of the distribution of CKS2 expression in patients with lymph node metastasis or not. **(B)** ROC curves of discriminating endometrial carcinoma patients with lymph node metastasis or not. T, tumor size; N, lymph node metastasis.

Supplementary Figure S3 Genetic alteration of CKS2 in endometrial carcinoma. The bar chart demonstrated the genetic alteration status of CKS2 in 548 endometrial carcinoma samples profiled in mRNA expression and protein expression.

Supplementary Figure S4 Potential upstream transcriptional regulators of CKS2. Network for predicted miRNAs (marked in blue squares) and TFs (marked in green diamonds).

Supplementary Figure S5 Functional annotations for genes negatively correlated with CKS2 in endometrial carcinoma. **(A)** Network of enriched terms colored by cluster ID. **(B)** Network of enriched terms colored by *p*-value.

## Abbreviations

AUC, area under the curve; CKS2, cyclin-dependent kinases regulatory subunit 2; DEG, differentially expressed gene; GEO, Gene Expression Omnibus; GO, gene ontology; GTEx, Genotype-Tissue Expression; HR, hazard ratio; IHC, immunohistochemistry; SMD, standard mean difference; SROC, summarized receiver operating characteristics; TCGA, The Cancer Genome Atlas; TF, transcription factor; TIMER, Tumor Immune Estimation Resource; TPM, transcripts per million.
