# Supplementary figures and images for: Expression Profile and Molecular Basis of Cyclin-Dependent Kinases Regulatory Subunit 2 in Endometrial Carcinoma Detected by Diversified Methods
Source: Pathol Oncol Res. 2022 May 27;28:1610307. doi: 10.3389/pore.2022.1610307 (PMC9184457; doi:10.3389/pore.2022.1610307)

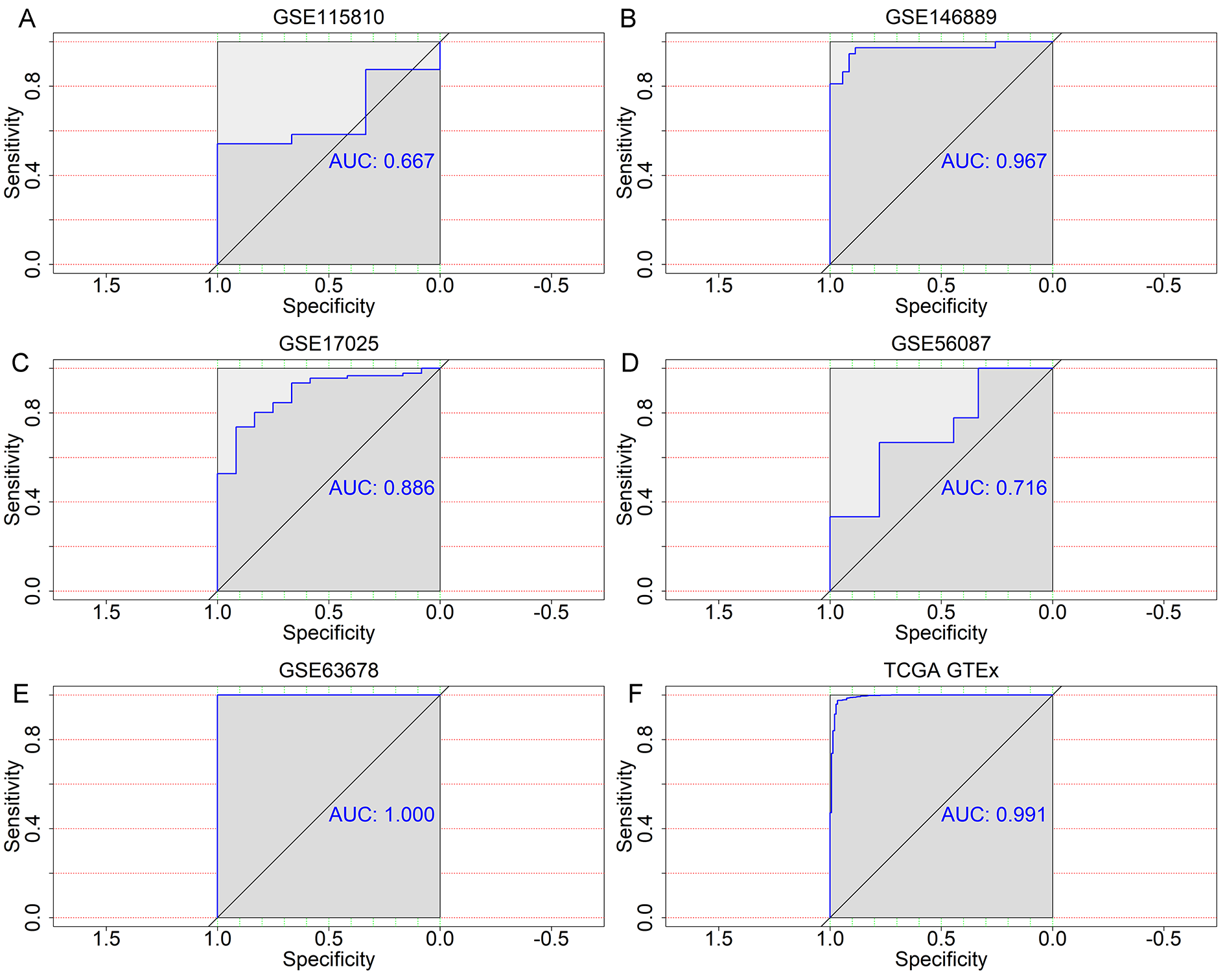

Supplement: Supplementary file 2 [file Image1.TIFF]

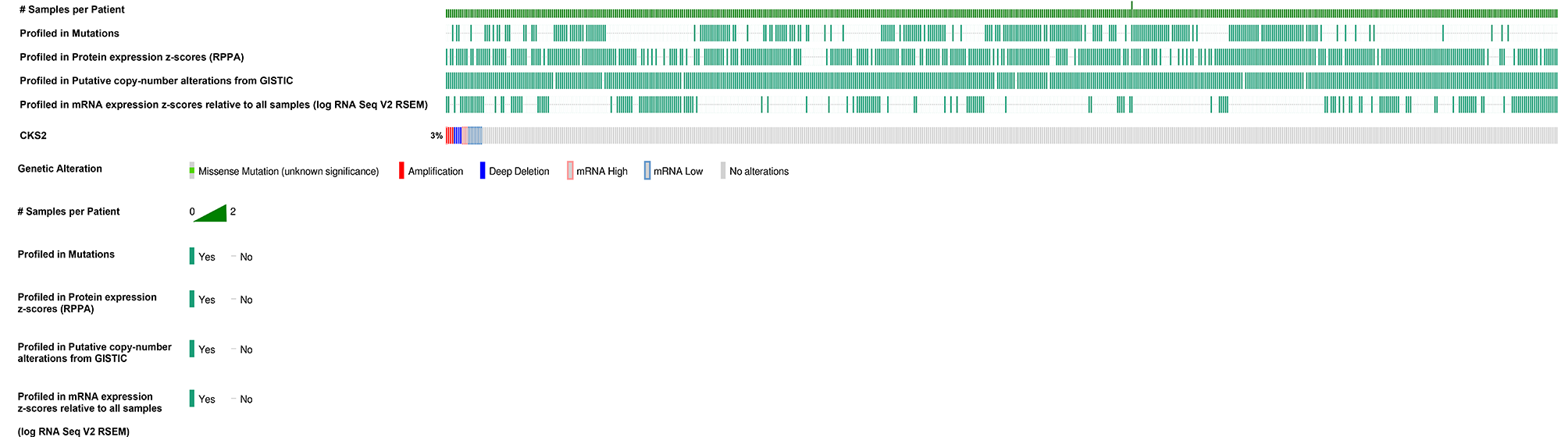

Supplement: Supplementary file 3 [file Image3.TIF]

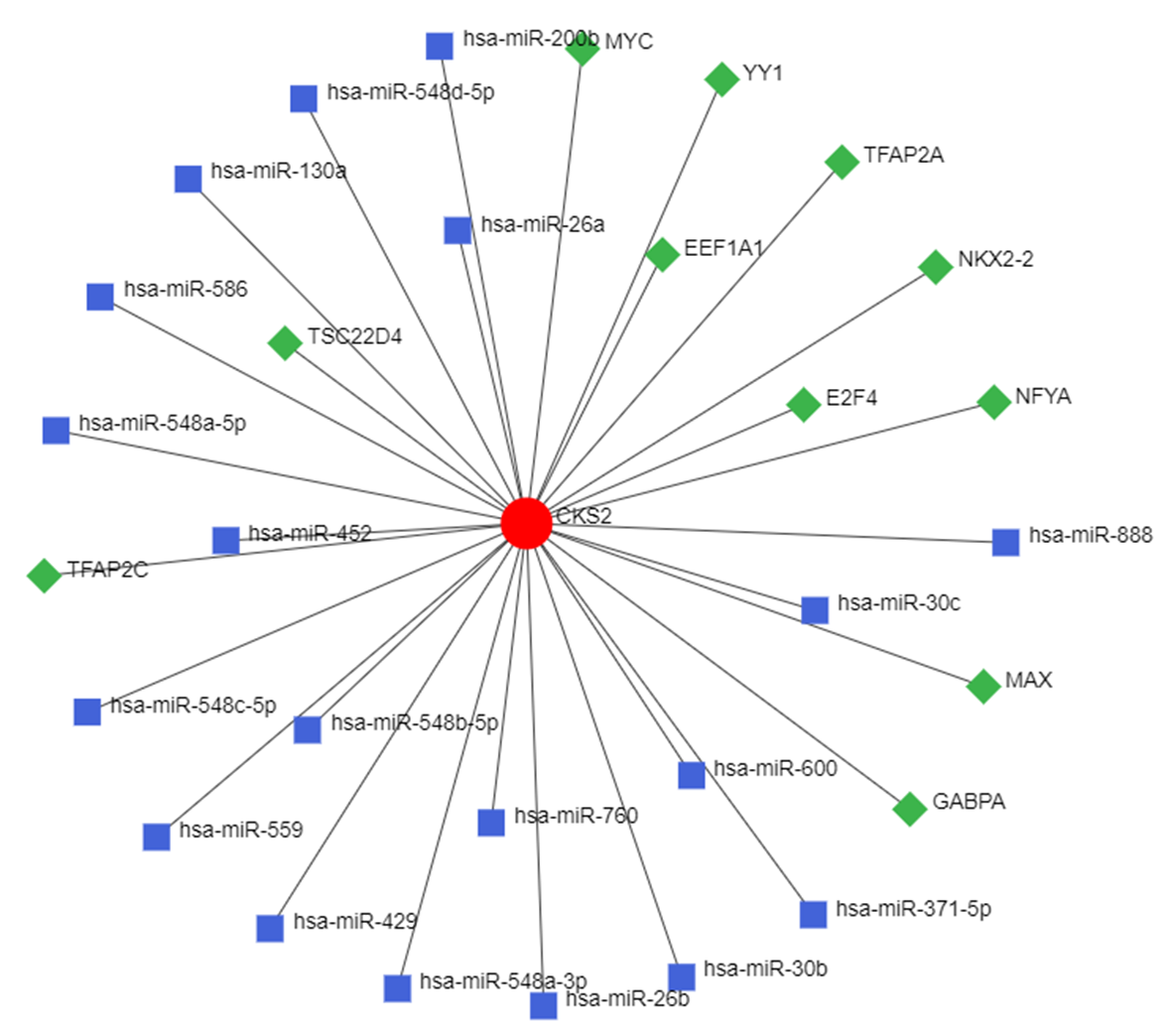

Supplement: Supplementary file 4 [file Image4.TIF]

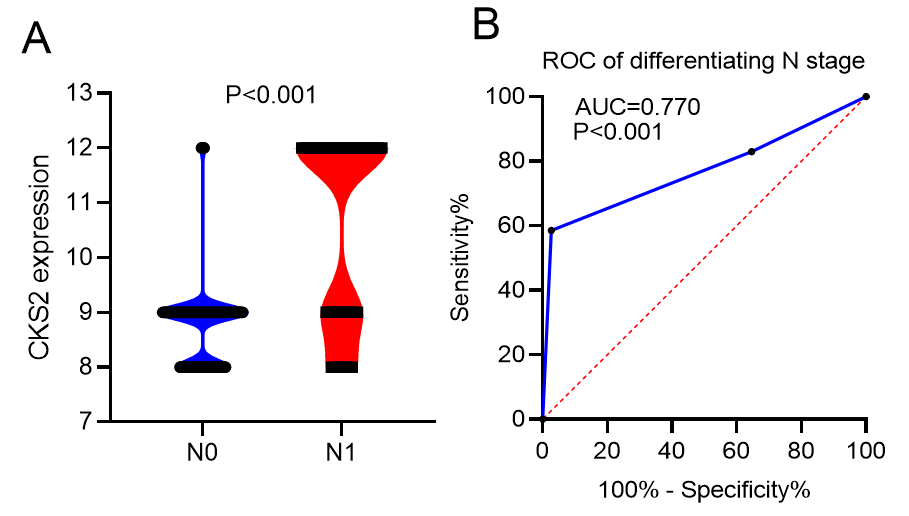

Supplement: Supplementary file 5 [file Image2.TIF]

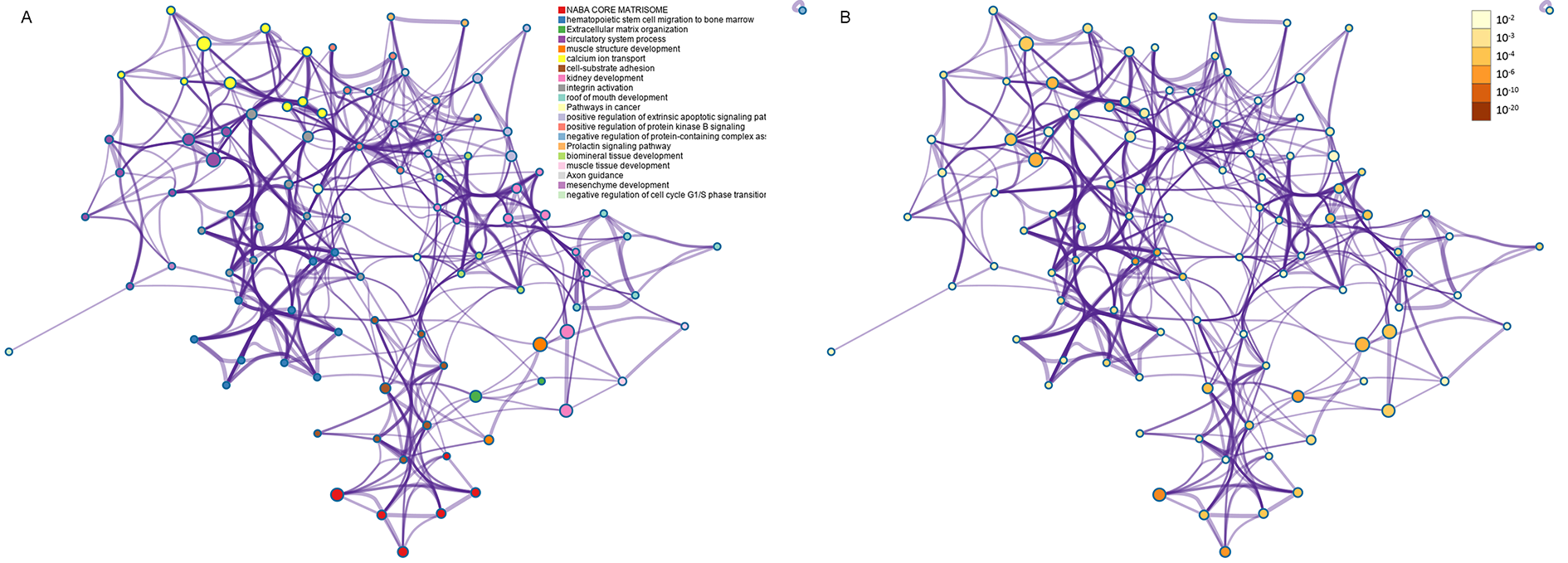

Supplement: Supplementary file 7 [file Image5.TIF]
